# Relationship between clinical and surgical findings and reparability of large and massive rotator cuff tears: a longitudinal study

**DOI:** 10.1186/1471-2474-15-180

**Published:** 2014-05-26

**Authors:** Richard Holtby, Helen Razmjou

**Affiliations:** 1 Holland Orthopaedic & Arthritic Centre, Sunnybrook Health Sciences Centre, 43 Wellesley Street East, Toronto, Ontario M1Y 1H1, Canada; 2 Department of Surgery, Faculty of Medicine, University of Toronto, Toronto, Canada; 3 Department of Physical Therapy, Faculty of Medicine, University of Toronto, Toronto, Canada

**Keywords:** Large, Massive, Rotator cuff tear, Partial repair, Predictors

## Abstract

**Background:**

The literature has shown good results with partial repairs of large and massive tears of rotator cuff but the role of factors that affect reparability is less clear. The purpose of this study was twofold, 1) to examine clinical outcomes following complete or partial repair of large or massive full-thickness rotator cuff tear, and 2) to explore the value of clinical and surgical factors in predicting reparability.

**Methods:**

This was a secondary data analysis of consecutive patients with large or massive rotator cuff tear who required surgical treatment (arthroscopic complete or partial repair) and were followed up for two years. Disability measures included the American Shoulder and Elbow Surgeons (ASES), the relative Constant-Murley score (CMS) and the shortened version of the Western Ontario Rotator Cuff Index (ShortWORC). The relationship between predictors and reparability was examined through logistic regressions and chi-square statistics as appropriate. Within group change over time and between group differences in disability outcomes, range of motion and strength were examined by student’s T-tests and non-parametric statistics.

**Results:**

One hundred and twenty two patients (41 women, 81 men, mean age 64, SD = 9) were included in the analysis. There were 86 large (39 fully reparable, 47 partially reparable) and 36 (10 fully reparable, 26 partially reparable) massive tears. Reparability was not associated with age, sex, or pre-operative active flexion or abduction (p > 0.05) but the fully reparable tear group showed a better pre-operative ASES score (p = 0.01) and better active external rotation in neutral (p = 0.01). Reparability was associated with tear shape (p < 0.0001), size (p = 0.002), and tendon quality (p < 0.0001).

**Conclusions:**

Reparability of large or massive tears is affected by a number of clinical and surgical factors. Patients whose tears could not be fully repaired showed a statistically significant improvement in range of motion, strength and disability at 2 years, although they had slightly inferior results compared to those with complete repairs.

## Background

There are many factors that affect recovery following rotator cuff repair. Among those are the patient’s demographics, pre-operative level of disability, clinical presentation, imaging such as humeral head position, fatty infiltration and tear size and surgical findings such as tear size and shape and tendon quality and retraction. The relationship between size of the rotator cuff tear and recovery based on patient oriented outcomes remains controversial. While some authors point out that tear size is not an important contributor to outcomes or overall satisfaction with surgery [1-6], others [7-10] report that larger tears have a less predictable recovery of strength and function and are associated with more residual disability.

The relationship between tear reparability and recovery is also a subject of debate. The limited literature has shown that partial repairs, sometimes referred to as functional repairs, provide surprisingly good results despite incomplete closure of the defect of the cuff [11-15]. The concept of functional repair was first introduced by Burkhart and colleagues in the early 1990’s [2,16-18]. The authors proposed that partial repairs would restore the force couple of the humeral head and increase acromion-humeral distance, resulting in dramatic changes in pain and function [16-19]. Consistent reports of successful results of partial repairs is promising but the failure to demonstrate superiority of complete repair over partial repair in the limited literature might be due to lack of statistical power in studies that have used small sample sizes of patients [13]. Improved life style of the aging population over the past decades demands better function in these individuals, which increases the need for managing larger and more disabling rotator cuff tears. Therefore, investigating factors that affect reparability of a cuff tear and their interaction with disability can help clinicians to choose surgical candidates more accurately.

The purpose of this study was therefore twofold, 1) to examine clinical outcomes following complete or partial repair of large or massive full-thickness rotator cuff tear, and 2) to explore the value of clinical and surgical factors in predicting reparability. We hypothesized significant improvement in pain and function in both partial and complete repair groups. The second hypothesis was that pre-operative active external rotation, strength, and intra operative findings such as size, shape and tendon quality would affect reparability.

## Methods

This was a secondary data analysis of consecutive patients with large or massive rotator cuff tears who required surgical treatment (full or partial repair) and were followed up for two years. Patients with isolated subscapularis tears were excluded. Standardized clinical and surgical data and disability scores had been collected prospectively and entered into a shoulder research database. All patients were immobilized in an Ultrasling for 6 weeks and followed a standardized rehabilitation protocol. Patients had provided written informed consent for participation in previous studies. Approval for using the existent database was obtained from the Research Ethics Board of the Sunnybrook Health Sciences Centre.

### Patient-related outcome measures

Two joint specific outcome measures, the American Shoulder and Elbow Surgeons (ASES) Score [20], and the relative Constant-Murley score (CMS) [21], and a condition specific outcome measure, the shortened version of the Western Ontario Rotator Cuff Index (ShortWORC) [22] were used to document change in disability over a period of two years (within group difference) and to investigate differences in disability between the full and partial repair groups (between group differences). All outcomes have been reported to be valid and reliable in patients with rotator cuff pathology [22-26]. Level of co-morbidity (0-52) was calculated as continuous data based on a validated score, the Cumulative Illness Rating Scale [27] which examines the overall health. In this scale, zero represents no impairment and a score of 52 represents the highest level of possible impairment.

### Clinical predictors

Clinical examination of the affected shoulder included active range of motion (ROM) in flexion, abduction and external rotation in neutral position. To further categorize weakness, active flexion was then categorized as pseudoparalysis (being less than 100°) and no pseudoparalysis in the presence of full passive forward flexion. Strength was measured as the maximum force that the patient could resist for 5 seconds without significant pain and discomfort at approximately 60-90° of elevation and in the scapular plane with an unsecured tensiometer.

### Surgical predictors

All arthroscopic surgical procedures were performed by one surgeon with 30 years of experience in shoulder reconstruction. Surgery was performed with the patient under general anesthesia in the lateral decubitus position. After inspection of glenohumeral joint structures, the tear size and shape and tendon quality were documented. The medial-to-lateral and anterior-to-posterior dimensions of the tear were measured arthroscopically using a calibrated probe. Size of rotator cuff tear (largest dimension) was categorized as large (>3-5 cm.) and massive (5 cm. and larger) [28]. The tear shape was classified as crescent, L-shaped, reverse L-shaped, and U-shaped. The crescent tears were short and wide, where L-shaped tears were long and narrow resembling a normal or inverted L. The U-shaped tears were longitudinal and wide and resembled a U [29]. Margin convergence or side to side techniques along with lateral suture anchors were used when possible to repair the rotator cuff to the bone by single-row or double-row fixations.

Tear quality was based on the thickness of the cuff and it's friability as defined by the ease with which the free edge can be debrided with an arthroscopy shaver and the ability of the cuff to hold the stitches that are put in it. The mobility of the tendon was examined using an arthroscopic grasper through an accessory lateral portal and the distance from the greater tuberosity that the tear edge can be pulled to and was classified as “mobilized to anatomical position” or “number of centimeters medial to the greater tuberosity”. Partial repair applied to those tears that had a residual defect of more than 1 cm. Complete repair was subcategorized as 1) anatomical repair and 2) repair over the articular margin with less than 1 cm lateral residual gap.

### Statistical analysis

The relationship between clinical and surgical findings and reparability was examined through logistic regression and chi-square statistics as appropriate. Potential interactions between variables were explored as required. Within group change over time and between group differences in pre and post-operative disability scores, active ROM, and strength levels were examined. The difference in the amount of change (post-pre scores) were also examined by independent student’s T-tests and non-parametric statistics depending on normality of the data.

## Results

One hundred and twenty two patients (41 women and 81 men, mean age 64, SD = 9, range 40-90) were included in the analysis. There were 86 large (39 fully reparable, 47 partially reparable) and 36 massive (10 fully reparable, 26 partially reparable) tears. Overall, 49 patients had a full repair and 73 had a partial repair. There were 26 crescent-shaped, 26 L-shaped (18 L-shaped, 8 reversed), and 70 U-shaped tears. In the complete repair group, 23 (45%) had an anatomical repair and 26 (53%) had less than 1 cm lateral defect.

### Reparability and patient demographics

Table 1 shows demographics of the groups. Both groups were comparable with respect to age, sex, comorbidity, hand dominance, side of surgery, symptom duration, incidence of night pain, incidence of trauma, having a work-related injury and smoking habits (p > 0.05). Biceps pathology was distributed evenly between groups.

**Table 1:** Descriptive data of 122 patients (partial repair vs. complete repair)

**Variables**	**Partial repair (73)**	**Full repair (49)**	Statistics*
**Age** (years)	67, SD = 9 (40-90 y)	64, SD = 9 (41-83 y)	p = 0.09
**Symptom duration** (months)	42, SD = 61	50, SD = 58	p = 0.49
**Comorbidity (0-52)**	3.93, SD = 2	4.04, SD = 2	P = 0.80
**Smoker**	5 (7%)	3 (6%)	p = 1.00
**Sex**			
Male	48 (66%)	33 (67%)	p = 0.86
Female	25 (34%)	16 (33%)	
**Dominant side**			
L	2 (3%)	1 (2%)	P = 0.83
R	71 (97%)	48 (98%)	
**Affected side**			
L	21 (29%)	12 (23%)	P = 0.52
R	52 (71%)	37 (77%)	
**Mechanism of injury**			
Traumatic	22 (30%)	11 (22%)	P = 0.41
Non-traumatic	51 (70%)	38 (78%)	
**Work-related injury**	9 (12%)	6 (12%)	p = 0.99
**Night pain**	48 (65%)	32 (48%)	p = 0.96
**Tear size**			
Large	47 (64%)	39 (80%)	p = 0.07
Massive	26 (36%)	10 (20%)	
Largest dimension (cm)	4.43, SD = 0.87	3.96, SD = 0.90	p = 0.002
**Tear shape**			
Crescent	6 (8%)	20 (41%)	p < 0.0001
L-shaped	8 (11%)	18 (36%)	
U-shaped	59 (81%)	11 (22%)	
**Tendon quality**			
Good	6 (8%)	20 (41%)	p < 0.0001
Fair	59 (81%)	28 (57%)	
Poor	8 (11%)	1 (0.2%)	
**Biceps pathology**			
Full rupture	22 (30%)	15 (31%)	P = 0.98
Partial rupture	30 (41%)	20 (41)	
Subluxed/dislocated	4 (5%)	3 (6%)	
**Associated surgeries**			
Lat clavicle resection	29 (53%)	26 (53%)	p = 0.19
Biceps tenodesis	9 (12%)	5 (10%)	p = 0.78
Biceps tenotomy	2 (3%)	2 (4%)	p = 1.00
Debridement for OA	8 (11%)	2 (4%)	p = 0.31

*Fisher’s Exact Test or chi-square statistics were used for categorical data.

Although age and sex were not different between groups, they played an important role in tendon quality, size and shape of the tear. Age was correlated with tear size (p = 0.045), tendon quality (p = 0.001), and tear shape (p = 0.021) with older patients having slightly larger tears, poorer tendon quality and more U-shaped tears.

Sex-related differences were observed in some factors. Men had more traumatic injuries (84% vs. 51%) with women reporting more insidious onsets (p = 0.0001). Men had a slightly better tendon quality (25% of men fell in good category vs. 15% of women) but this was not statistically significant (p = 0.2). Men had more crescent-shaped tears (28% vs. 7%) than women. Women had more L-Shaped tears (32% vs. 16%), with the incidence of U-shaped tears being fairly similar between sexes (61% in women and 56% in men) (Fisher’s p = 0.01).

### Reparability and clinical findings

Reparability was not associated with pre-operative active flexion or abduction but the complete repair group showed a better active external rotation in neutral prior to surgery (p = 0.01). Incidence of pre-operative pseudoparalysis (active flexion of <100°) was not different between groups (p = 0.35). However, pseudoparalysis was more often observed in the massive tears (56% of massive tears had active flexion of less than 100 vs. 33% in the large tears, p = 0.02). Pseudoparalysis was more often observed in U-shaped tears (50% vs. 30%) than other tear shapes (p = 0.04). Table 2 shows the difference in pre and post-operative clinical and surgical findings between the fully reparable and partially reparable groups.

**Table 2:** Pre and post-op disability and range of motion scores of groups

**Variables**	**Partial repair 73**	**Full repair 49**	**Statistics (group differences)**
**Pre-operative scores**			
ASES (0-100)	42.69	51.05	p = 0.01
CMS (0-150)	44.03	47.64	p = 0.41
ShortWORC (0-100)	34.57	38.87	p = 0.17
Flexion	110.14	119.90	p = 0.16
Incidence of pseudoparalysis	31 (42%)	17 (34%)	p = 0.38
Abduction	102.67	107.18	p = 0.54
External rotation	36.16	44.39	p = 0.01
Strength	3.47	4.84	p = 0.11
**Post-operative scores**			
ASES (0-100)	71.42	82.82	p = 0.003
CMS (0-150)	73.73	87.92	p = 0.007
ShortWORC (0-100)	62.70	79.38	p = 0.003
Flexion	129.45	153.37	p = 0.002
Incidence of pseudoparalysis	18 (25%)	5 (10%)	p = 0.045
Abduction	121.25	142.45	p = 0.003
External rotation	42.81	49.06	p = 0.12
Strength	5.92	9.90	p = 0.001

### Reparability and surgical findings

Reparability was affected by tear shape (p < 0.0001) and size (p = 0.007), and tendon quality (p < 0.0001). Crescent tears were more amenable to full repairs as compared with U-shaped tears consistent with what Burkhart and colleagues have suggested [29]. The U-shaped tears have a larger mediolateral component which makes the convergence of tear edges more challenging. As expected, fully reparable tears were on average smaller than partially reparable tears when measured in cm as a continuous variable (p = 0.002). However, when tear size was examined on categorical basis as large and massive, the reparability was similar between sizes (p > 0.05). This is more of a statistical finding and points to the fact that the nature of variables (continuous vs. categorical) will affect the sensitivity of analysis. The majority of both groups had fair tendon quality (81% in partial repair vs. vs. 57% in the full repair). Only eight percent of the patients in the partial repair group fell in the good category vs. 41% in the complete repair group. When all factors were examined together (tendon quality and tear size and shape), shape of the tendon remained the only predictor of reparability in the sample studied.

### Reparability and disability scores

In terms of pre-op disability scores, the partial repair group had a higher disability score in ASES (43 vs. 51, p = 0.01) but not on other two outcomes (Table 2).

### Reparability and recovery

Post-operatively, the partial repair group reported higher residual disability scores as measured by all patient-oriented measures (ASES, RCMS and ShortWORC) and had lower mobility (flexion and abduction) and strength. However, post-op external rotation was not different between groups (Table 2). Number of patients with pseudoparalysis at two years was higher in the partial repair group (25% vs. 10%, p = 0.045).

Table 3 shows within group and between group differences in the amount of change. Both groups showed a statistically significant improvement in ASES, CMS, and ShortWORC, flexion, abduction and strength over 2 years. The complete repair group did not show a statistically significant change in active external rotation over time (p = 0.10). However, change in external rotation was significant in the partial repair group (p = 0.03), due to their more significant pre-operative weakness. Range of motion in all three directions showed similar amount of change in both groups. Strength had a better improvement in the full repair group.

**Table 3:** Post-operative minus pre-operative (change)

**Variables**	**Within group change**		**Between group differences**
	**Partial repair**	**Complete repair**	
ASES (0-100)	28.72 (p < .0001)	31.77 (p < .0001)	p = 0.53
CMS (0-150)	29.70 (p < .0001)	40.28 (p < .0001)	p = 0.03
ShortWORC (0-100)	28.13 (p < .0001)	40.51 (p < .0001)	p = 0.02
Flexion	19.32 (p = 0.001)	33.47 (p < .0001)	p = 0.23
Abduction	18.58 (p = 0.002)	35.27 (p < .0001)	p = 0.15
External rotation	6.64 (p = 0.03)	4.67 (p = 0.10)	p = 0.64
Strength	2.45 (p < .0001)	5.06 (p < .0001)	p = 0.003

## Discussion

Our study showed that higher subjective disability, limited active external rotation, poor tendon quality, larger tear size, and U-shaped tears were more associated with a partial repair. However, both partial and complete repairs had a statistically significant improvement in their disability level and range of motion. This is consistent with the early theories about rotator cuff pathology and its management proposed by Codman some 80 years ago [30]. In his work, he mentioned the potential benefit of partial coverage of the rotator cuff defect. In the early 1990s, Burkhart [16-18] coined the term functional tear of rotator cuff to describe a tear that was anatomically deficient but biomechanically intact. He hypothesized that by improving the transverse force couple between the anterior cuff (subscapularis) and the posterior cuff (infraspinatus and teres minor), the overall function of the shoulder joint can improve.

The limited literature [11-15,31,32] in this area has reported promising results for partial repairs of the rotator cuff which is consistent with our findings. In our study, the partial repair group showed a statistically significant improvement in range of motion, strength and disability level over a period of two years. Having a more obvious improvement in external rotation in the partial repair group is a new finding that supports Burkhart’s theory of improving the transverse force by providing more coverage of the cuff defect. Despite the dramatic improvement in clinical and disability scores, the partial repair group appeared slightly more disabled at 2 years when scores were compared cross-sectionally (post-op scores compared at one point). Similarly, comparison of the amount of change (post-op minus pre-op scores) showed less favorable results in the partial repair group in strength, CMS and ShortWORC scores.

A statistically significant improvement in external rotation of the partial repair group is a positive finding which is potentially related to a more significant weakness in external rotators. Wellmann et al, who examined a small sample of 24 patients with a large retracted rotator cuff tear found improvement in active flexion and abduction with a slight deterioration in external rotation [5]. This inconsistency may be related to differences in patient demographics, tear size or surgical techniques. However, since active external rotation appears to be a predictor of reparability, further examination of its significance is warranted.

Two studies have compared the outcomes of complete repair with partial repair [12,13,33]. Iagulli et al compared 45 patients with a complete repair to 41 patients with a partial repair and found no statistically significant difference in their UCLA at 2 years [13]. Kim et al reported similar outcomes between healed and unhealed groups of patients with a partial repair [33]. Another study [12] of 41 non-randomized patients with a partial repair (margin convergence) to a group of patients with a repair of the posterior interval slide edge showed no significant difference in range of motion and patient-related outcomes between groups. Superior results with a complete repair is expected as a complete repair is associated with better coverage of the humeral head and therefore better pain relief and improved function and the lack of difference found in the previous studies is potentially due to a lack of statistical power or type of outcome measure used.

In terms of clinical findings, the massive tear group showed a higher incidence of pseudoparalysis. However, active flexion of less than 100° was not a negative predictor of reparability. Consistent with our findings, Oh et al [34], reported that pseudoparalysis was not associated with poorer results in patients with large or massive tear. Unlike active flexion, better active external rotation was associated with a complete repair in our sample. Flexion is affected by a number of muscles, where external rotation in neutral is more specific to infraspinatus and would be more sensitive to pathology in the posterior cuff muscles.

In our study, tendon quality, and tear size and shape affected reparability. The crescent-shaped had the best outcome and U-shaped tears were more associated with a partial repair. The U-shaped tears have a larger mediolateral component which makes the convergence of tear edges more challenging. It has been reported that preoperative musculotendinous junction medialization, tendon retraction, and muscle quality are all predictive of tendon healing postoperatively when using a single-row rotator cuff repair technique [35]. Our study adds to the literature in this area as it explores the relationship between reparability and other non-surgical factor such as disability and clinical features used for clinical decision making.

In summary, failure to achieve a complete rotator cuff repair is multifactorial. Patient-reported disability and pre-operative clinical findings in conjunction with MRI investigations are potentially the most informative information for the clinicians. Intra-operative findings such as tear size, mobility, and shape are also critical but cannot be used to communicate the results prior to surgery. Future prospective studies are needed to incorporate the results of pre-operative MRI, the degree of weakness of the infraspinatus into clinical decision making process.

### Limitations

This study involved secondary analysis of data of patients with large or massive tears who had been followed up longitudinally. Due to retrospective nature of data analysis, role of preoperative imaging (e.g. fatty infiltration, humeral head position, tear retraction) was not investigated. In addition, no post-operative investigations had been performed to allow us to examine the repair integrity.

## Conclusions

Among demographics, clinical examination and patient-oriented outcomes, the pre-op scores of the ASES and external rotation in neutral position were predictive of having a partially reparable large or massive tear. Surgical variables associated with a partial repair were tendon quality and tear size and shape. Patients with partial repair had a statistically significant improvement in range of motion and strength and all disability scores over time. However, they had less flexion and abduction and reported a higher level of residual disability at 2 years post-operatively.

## Competing interests

The authors declare that they have no competing interests.

## Authors’ contributions

RH conceived the idea and performed the surgical procedures. HR supervised data collection and data entry. Both authors wrote the protocol and obtained Ethics Approval. HR conducted the statistical analysis and drafted the manuscript. Both authors have read and approved the final manuscript.

## Pre-publication history

The pre-publication history for this paper can be accessed here:

http://www.biomedcentral.com/1471-2474/15/180/prepub
